# Lifestyle choices mediate the association between educational attainment and BMI in older adults in China: A cross-sectional study

**DOI:** 10.3389/fpubh.2022.1000953

**Published:** 2022-10-26

**Authors:** Lu Wang, Jianxue Ren, Junli Chen, Runguo Gao, Bingyu Bai, Hongqing An, Weiqin Cai, Anning Ma

**Affiliations:** ^1^School of Management, Weifang Medical University, Weifang, China; ^2^School of Public Health, Weifang Medical University, Weifang, China; ^3^School of Nursing, Weifang Medical University, Weifang, China

**Keywords:** educational attainment, BMI, smoking amount, screen time, behavioral lifestyle, mediating effect

## Abstract

As the Chinese population ages, unhealthfully high body mass index (BMI) levels in older adults are becoming a public health concern as an unhealthfully high BMI is an ill-being condition and can contribute to the risk of disease. Education and lifestyle choices affect BMI; however, the evidence on the relationships and interactions among these factors remains unclear. This study aimed to investigate the mediating effect of lifestyle choices on educational attainment and BMI among older adults in China. Using the Chinese Family Panel Studies (CFPS) 2018 panel data, this study integrated personal- and family-level economic data libraries, including 7,359 adults aged ≥60 years. Lifestyle parameters included smoking amount and screen time. Height and weight values were used to calculate BMI. The chi-square test, binary logistic regression analysis, stepwise regression analysis, and bootstrapping mediating effect tests were used for data analysis. Single-factor chi-square test revealed differences in BMI levels among groups defined by sex, age, residence, marital status, per capita annual household income, education years, and lifestyle choices. Binary logistic regression showed that age, residence, education years, smoking amount, and screen time influenced BMI. Stepwise regression results showed that education years, smoking amount, and screen time were associated with BMI (*t* = 3.907, −4.902, 7.491, *P* < 0.001). The lifestyle variables had partial mediating effects on BMI. The mediating effect of lifestyle on BMI was 0.009, while smoking amount was 0.003, and screen time was 0.006. Unhealthfully high BMI levels are increasing among older adults in China and are affected by many factors. Lifestyle factors and educational attainment can interact, affecting BMI. Interventions should consider lifestyle factors and education attainment to help maintain healthy BMI and reduce unhealthfully high BMI incidence.

## Introduction

Unhealthfully high BMI levels are a major public health problem. China has the highest number of people with obesity worldwide (1–3). A 2020 report indicated that more than 50% of Chinese residents have overweight/obesity; specifically, 34.3% and 16.4% of residents aged ≥18 years have overweight and obesity, respectively (4). Obesity affects the health of the Chinese population (4), increasing the risk of disease and death (5, 6). Poor population health is associated with high disease management costs (3). Obesity is particularly harmful to older adults, among whom it co-occurs with other diseases and a general decline in body resilience (7). China has an aging population, making high BMI among older adults a public health concern.

The increase in obesity rates in China is associated with rapid economic growth, urbanization, and lifestyle changes (2). China's economy has undergone rapid growth in the past three decades, while the rate of urbanization has increased, affecting lifestyle choices and living standards. Lifestyle choices are a significant contributor to obesity (8). For example, lifestyle risk factors such as smoking, unhealthy diet (e.g., insufficient intake of vegetables and fruits, excessive intake of fat and sodium), lack of physical activity, sedentary behavior, and the prevalence of alcohol use all influence obesity (2, 8, 9). Behavioral interventions targeting these factors may help achieve weight loss and reduce obesity risks (10–12).

Educational attainment and obesity rates are linked (13, 14); this relationship may be mediated by socioeconomic status, with negative correlations commonly reported in high-income countries and positive correlations commonly reported in low-income countries (15, 16). Education attainment is associated with the extent of health knowledge and access to health-related resources, which can affect obesity rates (17). Some evidence suggests that education may help reduce the incidence of obesity and some obesity-related diseases (18), including among older adults. Older men and women with relatively low education levels are more likely to have obesity than their more highly educated counterparts (7). The impact of education level on obesity needs further research (19).

Education and health literacy levels are correlated (20), whereby people with greater health literacy tend to make better health choices (21) and are more concerned with the consequences of their choices (22). Overall, some evidence suggests that education may affect high BMI rates via lifestyle choices. Further research is required to validate these findings (23) and elucidate the interactions among these parameters.

The evidence on the interactions among education level, lifestyle choices, and BMI among older adults is scarce both in China and worldwide. This study aimed to examine these associations among Chinese older adults. This evidence can be used to design interventions aimed at reducing unhealthfully high BMI levels among older adults in China. Further, older adults in China as a cohort have a specific background. They have experienced extensive changes across a variety of eras, especially the major turning point of reform. Given their history, levels of education vary greatly among older adults in China. In their youth, China's compulsory education system was not yet universal. Therefore, in this cohort, some individuals received no education while others achieved higher education. Similarly, the concept, behavior, and lifestyle of older adults is different from other age groups. Therefore, it is of great significance to study the relationship between lifestyle and BMI among older adults.

## Materials and methods

### Data sources

Data for this study were obtained from the 2018 Chinese Family Panel Studies (CFPS) database. The CFPS is managed by the Institute of Social Science Survey, Peking University, for the purpose of tracking the population of 25 Chinese provinces (cities and districts, excluding Hong Kong, Macao, and Taiwan). It includes 95% of the Chinese population and is considered nationally representative. A stratified, multi-stage probabilistic sampling method proportional to the population size was used in this study. The survey aims to reflect the country's economic, social, demographic, health, and other types of changes.

### Data extraction

In the CFPS database, the database for personal includes the questionnaire data of all individuals aged ≥10 years, with a sample size of 32,669. The family economy database contains 14,241 samples, covering information related to family economy. This study screening of the 2018 CFPS person database and family economy database.

By integrating the variables of the two databases, a comprehensive database needed for research is formed. The variable extracted from the family economy database in this study were per capita annual household income (yuan). The reason for inclusion is that under the special social background of China, the living standard of older adults aged ≥60 largely depends on the level of family support. Except for the per capita annual household income (yuan), the other variables were obtained from the personal database.

#### Data categories and filters

In this study, we screened the 2018 CFPS person database and family economy database. First, 32,669 original samples of the personal database were screened according to the sample age, and 7,872 samples aged 60 and above were obtained. Other variables in this study included sex, age, height, weight, residence, marital status, per capita annual household income (yuan), education years, smoking amount per day, and screen time per week (h). Among them, the education years variable is a comprehensive education variable generated by the CFPS official according to the education information of different modules during the survey. Smoking amount per day is determined by the question that “How many cigarettes do you currently smoke on average per day?.” Screen time per week (h) was measured by the question that “How many hours per week do you spend watching TV, movies, and other video programs in various ways?.” Other variables were obtained by self-report and were screened one by one and some samples were excluded as inapplicable or missing data. The 88 missing values of education years were mainly excluded, and then BMI was calculated using height and weight data, and 210 missing values of BMI were excluded, followed by 25 missing values of smoking amount per day and screen time per week (h). A total of 190 missing values of the remaining research variables were excluded, and 7,359 final sample data were obtained. The smoking amount, and screen time were used as measures of lifestyle habits. Because adults aged ≥60 years tend to be retired or have reduced professional engagements, and with the decline of activities, such as work, the smoking amount, and screen times will more significantly influence their lifestyle, which affects body mass index (BMI) (4). Consequently, smoking amount and screen time were used as measures of lifestyle habits.

Height and weight were used to calculate BMI [BMI = weight (kg)/height (m^2^)]. The height and weight variables in this database were directly obtained from the statistical tracking results of the CFPS in 2018, and were reviewed and verified by the CFPS official to be reliable. According to the Chinese standard, BMI values of <24 kg/m^2^, 24 ≤ BMI <28 kg/m^2^, and BMI ≥ 28 kg/m^2^ represent underweight and normal weight, overweight, and obesity, respectively (4). According to the main purpose of this study, we mainly study the related conditions and influencing factors of unhealthfully high BMI, in older adults to determine the potential influencing factors and mediating factors of unhealthfully high BMI. Therefore, according to the Chinese standard of BMI values, BMI <24 was assigned as the non-high BMI group and BMI ≥ 24 the high BMI group, which represented overweight and obesity. The above two groups were analyzed with chi-square test and binary logistic regression to explore factors that may affect BMI. Based on previous studies, we classified variables required for the chi-squared test. Based on available data and previous reports, we classified education years as follows: 0–6, 7–9, 10–12, and >12 years. These groups correspond to primary school and below, junior high school, high school, and higher education, respectively. According to the original database information and the review of relevant data, the smoking amount among older adults who do not smoke is 0, 1–10 cigarettes is moderate smoking, 11–20 cigarettes is a large amount of smoking, and more than 20 cigarettes is heavy smoking. For the assessment of weekly screen time, this study evaluated the proportion of weekly screen time based on daily life patterns: 0 h indicates no screen time, 1–20 h relatively little weekly screen time and relatively healthy life, while 20–40 h is a relatively great screen time. More than 40 h was determined to be a huge amount screen time. In other analyses, in order to maximize the data validity of continuous variables, they were treated as continuous variables without classification.

### Statistical analyses

Single factor analysis was performed with the chi-square test. Binary logistic regression analysis was used to explore factors affecting BMI. Binary logistic regression analysis with the forward method was used to estimate odds ratios (*OR*) and 95% confidence intervals (*CI*) for variables. The *p*-value was truncated according to the inclusion criterion of 0.05 and the exclusion criterion of 0.10. Regression coefficients were estimated.

Mediating effects were evaluated based on the methods reported by Wen et al. (24). The bootstrap method was used to evaluate intermediary effects, starting with estimates of the c-coefficients, which were then assessed as indicators of mediating or masking effects. Subsequent tests were performed regardless of c-coefficient significance and involved stepwise analyses of coefficients a and b. The significance of both was considered indicative of an indirect effect, leading to the fourth step of the analysis. Otherwise, step three was implemented. The third step was to use the Bootstrap method to test H_0_: ab = 0. If the result was significant, the indirect effect was deemed significant, and then the fourth step is carried out. Otherwise, the indirect effect is not significant, and the analysis was stopped. Significant results at this step indicated an indirect effect, allowing the fourth step to be undertaken; when an indirect effect was non-significant, further analyses were not performed. The fourth step was to test the c'-coefficient; a non-significant c'-coefficient indicated a mediation effect only, leading to step five, which compared the a, b, and c' coefficients for evidence of partial mediating effects.

According to the above methods, stepwise regression analysis was used to examine the mediating effect of lifestyle factors and educational attainment on BMI. Mediating effect analysis used in this study was the parallel multiple mediating effect analysis that used model 4. This method can analyze the mediating effect of two or more mediating variables, respectively, and compare the mediating effect, etc. Compared with the simple mediating model with a single mediating variable, it can reflect the problem we want to discuss more comprehensively. The bias-corrected percentile bootstrap (5,000 repeated samples) method was used to test the mediating effect of lifestyle at α = 0.05.

In this study, STATA software was used for data screening, and IBM SPSS Statistics 24 software was used for statistical analyses.

## Results

### Chi-square analysis of BMI among Chinese older adults

The rate of Chinese older adults with BMI ≥ 24 was 37.2%. This rate is higher among females (38.9%) than among males (35.5%). Additionally, in the age group of 60–69 years, the rate of BMI ≥ 24 was 39.0%. This rate was among older adults who live in urban areas was 42.4%, which was higher than in rural areas. Divorced older adults had higher rates of BMI ≥ 24 (41.2%) than their married counterparts (38.1%). This rate increased with higher income and peaked at 51.2% at the income level of 75,000 and 99,999 yuan; the corresponding value for income levels ≥100,000 yuan was 44.1%. The prevalence of BMI ≥ 24 among those with 10–12 education years (high school and below) was the highest at 42.9%. The rate of BMI ≥ 24 among non-smokers was 40.1%. Longer weekly screen times increased BMI ≥ 24 risk. Older adults reporting 21–40 h of screen time per week had BMI ≥ 24 rates of 42.4% (Table 1).

**Table 1:** BMI among older adults in China (comparisons were made with the chi-square test; *n* = 7,359).

	**BMI <24**	**BMI ≥24**	**Chi-square value**	***P***
	***N* (%)**	***N* (%)**		
**Sex**				
Male	2,428 (64.5)	1,334 (35.5)	9.587	<0.01**
Female	2,196 (61.1)	1,401 (38.9)		
**Age (years)**				
60–69	2,906 (61.0)	1,859 (39.0)	32.840	<0.001***
70–79	1,369 (64.6)	750 (35.4)		
≥80	349 (73.5)	126 (26.5)		
**Residence**				
Urban	2,054 (57.6)	1,515 (42.4)	82.838	<0.001***
Rural	2,570 (67.8)	1,220 (32.2)		
**Marital status**				
Spinsterhood	51 (82.3)	11 (17.7)	21.227	<0.001***
Married	3,812 (61.9)	2,343 (38.1)		
Cohabiting	16 (64.0)	9 (36.0)		
Divorce	40 (58.8)	28 (41.2)		
Widowed	705 (67.2)	344 (32.8)		
**Per capita annual household income (yuan)**				
<25,000	3,469 (66.0)	1,785 (34.0)	82.944	<0.001***
25,000–49,999	722 (55.4)	581 (44.6)		
50,000–74,999	255 (55.1)	208 (44.9)		
75,000–99,999	79 (48.8)	83 (51.2)		
≥100,000	99 (55.9)	78 (44.1)		
**Education years**				
0–6	3,340 (64.7)	1,825 (35.3)	25.919	<0.001***
7–9	810 (59.3)	555 (40.7)		
10–12	373 (57.1)	280 (42.9)		
>12	101 (57.4)	75 (42.6)		
**Smoking amount per day**				
0	3,121 (59.9)	2,090 (40.1)	74.025	<0.001***
1–10	677 (72.9)	252 (27.1)		
11–20	684 (68.7)	312 (31.3)		
>20	142 (63.7)	81 (36.3)		
**Screen time per week (h)**				
0	605 (70.7)	251 (29.3)	45.029	<0.001***
1–20	2,904 (63.5)	1,668 (36.5)		
21–40	953 (57.6)	702 (42.4)		
>40	162 (58.7)	114 (41.3)		
**Total**	4,624 (62.8)	2,735 (37.2)		

*P <0.05; **P <0.01; ***P <0.001.

Single-factor chi-square test revealed differences in BMI levels among groups defined by sex, age, residence, marital status, per capita annual household income, education years, and lifestyle choices (Table 1). Considering the limitations of the chi-square test itself and the possible confounding factors in the inclusion of variables in this study, the results of the chi-square test only provide the basis and reference for subsequent research, and do not provide the final conclusion for this study. Based on the results of the single factor chi-square test, this result suggests that these factors influence BMI in Chinese older adults.

### Factors affecting BMI

After a binary logistic regression model was established, the omnibus test of model coefficients were performed (χ^2^ = 221.956, *P* < 0.001), suggesting that the overall model was statistically significant. The Hosmer and Leme showed test results suggested good model fit (χ^2^ = 11.904, *P* = 0.156). Age, marital status, and smoking amount were risk factors for BMI ≥ 24; urban residence, higher education years, and reduced screen time were protective factors for BMI ≥ 24 (Table 2).

**Table 2:** Binary logistic regression analysis results of factors affected of BMI in older adults in China.

**Independent variable (control group)**	***B***	***SE***	**Wald χ^2^**	***P***	***OR***	**95%*CI***
Age	−0.029	0.004	46.969	0.000***	0.971	0.963–0.980
Marital status (Widowed)			8.974	0.062		
Spinsterhood	−0.867	0.343	6.398	0.011*	0.420	0.214–0.823
Married	0.081	0.075	1.161	0.281	1.085	0.936–1.258
Cohabiting	0.149	0.427	0.122	0.727	1.161	0.503–2.679
Divorce	0.048	0.261	0.034	0.854	1.049	0.629–1.750
Residence (Rural)						
Urban	0.373	0.051	53.829	0.000***	1.452	1.314–1.604
Education years	0.016	0.006	7.895	0.005**	1.016	1.005–1.027
Smoking amount/day	−0.018	0.003	38.576	0.000***	0.982	0.976–0.987
Screen time/week	0.012	0.002	35.441	0.000***	1.012	1.008–1.016
Constant	1.044	0.313	11.153	0.001**	2.841	

*B* stands for regression coefficient; *SE* stands for standard error; The Wald χ^2^ and *P*-values correspond to hypothesis tests expressed as *B* coefficients; *OR* stands for odds ratio; 95% *CI* stands for 95% confidence interval.

*P < 0.05; **P < 0.01; ***P < 0.001.

### Mediating effects

The variables included in stepwise regression analysis are presented in Table 3. Model 1 indicates the directing effect of education years on BMI, model 2 indicates the direct effect of education years on smoking amount, model 3 indicates the direct effect of education years on screen times, while model 4 indicates the parallel multiple mediating effect of independent variable education years on BMI when smoking amount and screen time were included as two mediating variables (Table 3).

**Table 3:** Regression analysis results of smoking amount, screen time, and education years on BMI of older adults.

**Predictive variable**	**Model 1**		**Model 2**		**Model 3**		**Model 4**	
	**β**	***t***	**β**	***t***	**β**	***t***	**β**	***t***
Education years	0.048	4.798***	−0.102	−4.642***	0.246	7.150***	0.039	3.907***
Smoking amount							−0.026	−4.902***
Screen time							0.025	7.491***
*R* ^2^	0.033		0.192		0.044		0.042	
*F*	41.415***		291.615***		55.882***		40.659***	

*P < 0.05; **P < 0.01; ***P < 0.001.

**Figure 1:**
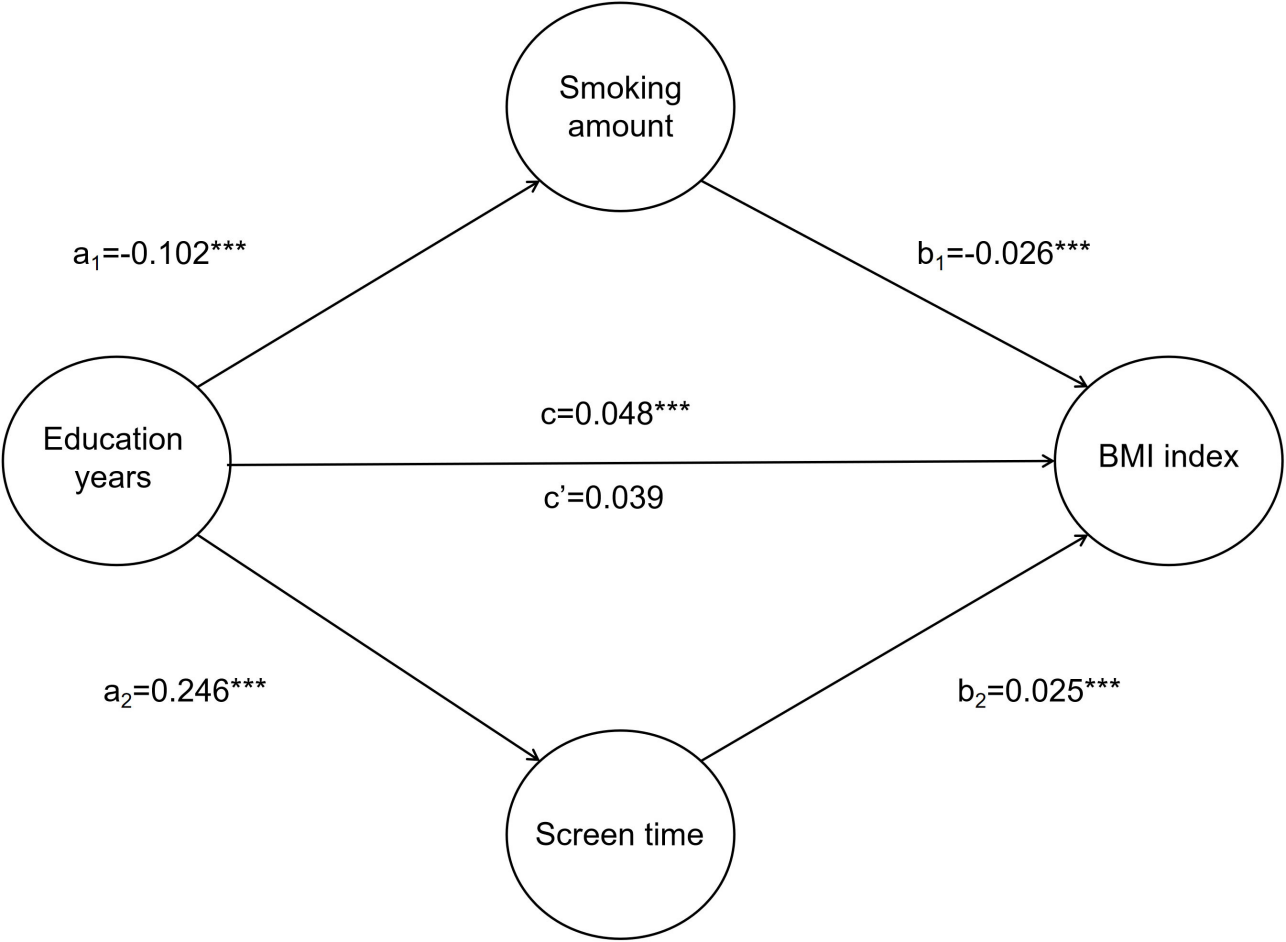
Mediating effect of smoking amount and screen time on education years and BMI. a_1_ represents the effect value of education years on smoking amount; a_2_ represents the effect value of education years on screen time; b_1_ represents the effect value of smoking amount on BMI index; b_2_ represents the effect value of screen time on BMI index; c represents the total effect value of education years on BMI index; c' represents the direct effect value of education years on BMI index. *P < 0.05; **P < 0.01; ***P < 0.001.

After controlling for demographic variables, education years affected smoking amount, screen time, and BMI (*t* = −4.642, 7.150, 0.048, *P* < 0.001). When the mediating variables, smoking amount and screen time, were included, smoking amount, screen time, and education years all affected BMI (*t* = −4.902, 7.491, 3.907, *P* < 0.001), and the partial regression coefficient associated with education years decreased from 0.048 to 0.039. The estimated direct effect is 0.039, lower than the total effect value of 0.048. Coefficients a_1_, b_1_, and c were the same symbol as a_2_, b_2_, and c, suggesting that the mediating effect of lifestyle choice on education level and BMI is a partial mediator, that is, both the direct effect and the mediating effect were present. Education years had a direct impact on BMI, but also an impact on BMI through the intermediary variable, namely behavioral lifestyle (Figure 1).

### Bootstrap mediating effect tests

The non-parametric percentile bootstrap test was used to test the hypothesis of the partial mediating effect of smoking amount and screen time on education years and BMI. The resulting CI did not contain zero, suggesting a partial mediating effect of smoking amount and screen time on the influence of education years on BMI (Table 4).

**Table 4:** Bootstrap tests of mediating effect between smoking amount, screen time, education years, and BMI in older adults.

	**Coeff**	**Boot *SE***	**Boot *LLCI***	**Boot *ULCI***
Total effect	0.048	0.010	0.028	0.067
Direct effect	0.039	0.010	0.019	0.059
Parallel multiple mediating effects	0.009	0.002	0.006	0.012
Smoking amount	0.003	0.001	0.001	0.004
Screen time	0.006	0.001	0.004	0.009
Difference	−0.004	0.001	−0.007	−0.001

The difference value reflects the difference between effect size values of smoking status and screen time.

## Discussion

The purpose of this study was to investigate whether lifestyle factors mediate the association between education attainment and BMI. The evidence for these relationships is complex, providing preliminary insights into the mediating effects of lifestyle on the relationship between education and BMI.

The rate of BMI ≥ 24 among older adults in China is 37.2%; this estimate is consistent with that recently reports (25) and is alarming (9, 26, 27). The prevalence rate of BMI ≥ 24 in this group is affected by sex, age, residence, marital status, per capita annual household income, years of education, and lifestyle choices. The obesity epidemic translates into a chronic disease epidemic in China and worldwide (28). The Chinese government and other organizations have attempted to control and prevent the increase in obesity and chronic disease rates in China (29). Evidence on the root causes of the increases in the rate of BMI ≥ 24 is required to develop effective prevention and management strategies.

The risk of BMI ≥ 24 in older adults in China increases with age. Older age is associated with metabolic changes in the body, including gastrointestinal absorption and digestive function decline, and heat absorption reduction. Concurrently, the risk of disease increases; some diseases may affect body tissue composition and increase the risk of malnutrition and body wasting (30). Smoking negatively affects the risk of BMI ≥ 24. Epidemiological and empirical studies have described an inverse relationship between smoking or nicotine use and body weight (27, 31). Thus, long-term exposure to nicotine may prevent excess weight gain; meanwhile, weight loss or management tends to be the causes of continued smoking (32). The rate of BMI ≥ 24 among older adults who live in urban areas is higher than in rural areas. Urban lifestyles are considered obesogenic due to high fat intake and low physical activity levels (33). Education level may protect against BMI ≥ 24. The rate of BMI ≥ 24 was the highest among older adults with 10–12 years of education; it decreased slightly among those with ≥12 years of education. Higher education may be associated with better quality of life, which reduces the risk of obesity (34). It is also associated with greater health literacy, which may enable better lifestyle choices and greater awareness of the associated consequences (7, 17). Individuals with high health literacy tend to be aware of the harm associated with obesity and may be more proactive in pursuit of habit change (35). The results of this study show that increased screen time is a risk factor for the occurrence of BMI > 24 and promotes increases in BMI. Increased screen time is associated with a sedentary lifestyle, which increases the risk of obesity (36, 37); among older adults, screen time is a risk factor for obesity (38, 39).

Lifestyle choices reflect older adults' attitude to health (8, 9). For most older adults, educational attainment and socioeconomic status tend to be stable variables; consequently, the association between educational attainment and obesity follows a relatively fixed pattern (40). We chose to evaluate smoking amount and screen time as indicators of lifestyle choices, which mediate the relationship between educational attainment and BMI, which is an indicator of overweight/obesity (39). Older adults should be encouraged to increase their health literacy, maintain their cognitive fitness, understand the relationship between lifestyle and obesity, and adjust their lifestyle choices, including exercise levels, to help maintain health (41). Population-level interventions should aim at achieving smoking cessation, moderate alcohol consumption, healthy diets, and increased physical activity, among others (42), helping older adults establish health-promoting habits. Community-level interventions should include fitness activities for older adults, such as square dancing and art clubs, reducing sedentary lifestyles and screen time among older adults. Through the above measures, the elderly are encouraged to maintain a reasonable BMI, thus promoting physical health.

This study had some limitations, which may affect the validity of our findings. First, the CFPS is a national, large-scale, multidisciplinary social tracking survey, which includes, but is not limited to, older adults. Therefore, there may be some limitations in the representation of older adults. Second, this study was based on cross-sectional data. Cross-sectional data are subject to omitted variable bias, where individual, unobserved effects may be associated with the observed variables. Consequently, the presented values may be over- or underestimates of the true effects. Given the available data, we used smoking amount and screen time to evaluate lifestyle factors. However, this study did not include positive behavioral variables. This may affect the results of our study to a certain extent by exaggerating the influence of smoking amount and screen time, namely, poor lifestyle habits, on unhealthfully high BMI levels, among older adults in China, while ignoring the mechanism of benign lifestyles on BMI. However, there is no doubt that smoking amount, as an important factor of bad behavior habits, and the length of screen time, as an important factor in measuring the degree of sedentary behaviors, both play a mediating role in the mechanism of BMI among Chinese elderly adults by education years, and also provide a basis for the control of BMI. Future studies should evaluate the impact of positive behaviors on BMI. Finally, some studies have shown that the feedback effects between the mediator and dependent variable will lead to simultaneity bias (43), which may have affected the presented results.

## Conclusion

This study examined the mediating impact of lifestyle choices on the relationship between educational attainment and BMI in older adults in China. The present findings may contribute to the development of prevention and management policies for unhealthfully high BMI levels. The BMI among older adults in China is alarming and is also affected by a variety of factors, including lifestyle choices and education levels. This evidence suggests that national-, community-, and individual-level interventions should be multifaceted to promote healthy choices among older adults in China, helping reduce unhealthfully high BMI levels in this population.

## Data availability statement

Publicly available datasets were analyzed in this study. This data can be found here: http://www.isss.pku.edu.cn/cfps/.

## Author contributions

WC and AM conceived the idea, organized the data analysis, and revised the manuscript. LW wrote the original draft preparation and visualization. JR and JC took part in data curation. RG, HA, BB, and WL participated in the discussion and revision of the manuscript. All authors have contributed to the final version of this manuscript.

## Funding

This study was funded by the Youth Program of the National Natural Science Foundation of China (grant number 72104186, 72004165), the Soft Science Project of Shandong Province Key Research and Development Program (grant number 2020RZB14001), the Humanities and Social Science Research Youth Fund program of the Ministry of Education (grant number 20YJCZH002), and the Natural Science Foundation Program of Shandong Province (grant number ZR2021MG019).

## Conflict of interest

The authors declare that the research was conducted in the absence of any commercial or financial relationships that could be construed as a potential conflict of interest.

## Publisher's note

All claims expressed in this article are solely those of the authors and do not necessarily represent those of their affiliated organizations, or those of the publisher, the editors and the reviewers. Any product that may be evaluated in this article, or claim that may be made by its manufacturer, is not guaranteed or endorsed by the publisher.
